# Annotations

**Published:** 1889-11-02

**Authors:** 


### Annotations.

**"Foreign Medical Literature."**

We propose in future to devote once in the month that part of our pages which we have called the "Practitioner's Outlook" to a review of the progress of medicine, surgery, therapeutics, and, to a limited extent, of forensic medicine and public health on the continent of Europe. As we have hitherto, in our abstracts of current English, so we shall in the foreign medical literature always endeavour to bear in mind the motto of *ne quid nimis*, and to confine our attention to points of practical interest to the busy practitioner in his daily work, avoiding such as concern only the scientific investigator or the specialist; in short, to adhere closely to the clinical side of every question. Good work is undoubtedly being done by individuals in every quarter of the globe, and we shall not fail to recognise it, but, practically, our field will be limited to France, Germany, Austria, Italy, and we may add Russia. Little or nothing can be hoped for from Spain and Portugal or their colonies, where, though the Sangrados are indeed extinct, and Dr. Da Cimba Bellem, of Lisbon, commanded a respectful attention at the Medical Congress in London, the science and art of medicine are what they were here fifty years ago, saving a faint reflection of modern research without its method and critical spirit. The practitioners of Scandinavia, not a whit behind their brethren of the other Teutonic races, are sadly handicapped by the limited opportunities afforded by the sparsity of the population, though they contribute occasionally to the German medical press, who, in common with Dr. Felix, of Bucharest, and other leading men of the Balkan States, received their professional education in Austria or Germany. France, it cannot be denied, has, in the scientific no less than in the political world, fallen from her high estate, though while Charcot, *facile princeps* among neurologists, with his colleagues at Les Salpetrière, Guerin, Béclard, Le Fort, Fournié, Cornie, Dujardin-Beaumetz, Javal, Ménière, Bronardel, and many other equally well-known names remain, she will still maintain her position as one, though not the first, among the great powers. Vain of their past reputation, they are too apt to betray an inexcusable ignorance of and indifference to the labours and discoveries of workers in other lands, forgetful of the fact that in these days of intellectual activity a knowledge of what is being done abroad is an essential condition of progress, and that *fas est ab hoste doceri*, even if we admit that such a hostile relation can exist in the world of science. The unmerited attention paid of late to hypnotism and suggestion, as well as extravagant assumptions and hopes indulged in by the disciples of Pasteur, are the natural outcome of the instability of the national character. Very different is the tone of Italian literature and work. The current medical journalism displays a surprising acquaintance with and appreciation of that of other countries, so much so, indeed, as frequently to constitute an Italian pamphlet a valuable bibliography of its subject. The *perfervidum ingenium* of the nation has seized on the most recent researches in bacteriology with excessive eagerness, and we see the almost amusing spectacle of municipal bodies gravely discussing bacilli and micrococci while neglecting the weightier matters of sewerage and water supply. But we believe that this is only the error of an intelligent people suddenly awakening to a renewed national life, fully aware that they must appropriate the entire accumulated intellectual wealth of the civilised world if they are to take their place by the side of others who have been longer in the field. In one department, however, the Italians can already more than hold their ground, though the brilliant surgeon Loreta is no more—Loreta, whose abdominal operations some of them never attempted before. There are not a few scarcely inferior to him, as Carle and the better known Porro, Apostoli, Mazzoni, and Bacelli, men who hold a position second to few, if any, in clinical medicine, and Ruate in both therapeutics and hygiene. The receptiveness of the Italian character, combined as it is with great capability of original research and intellectual acumen, gives promise of a rich harvest in the near future. German work is too well known to need description or commendation here; in every department of chemical, biological, or medical science the names of German *savants* and practitioners are household words. Nowhere does the laboratory hold such a place in the education of the student or the work of the hospital as there, and exhaustive, laborious, and accurate method is characteristic of German research and practice. Whatever the German's hand findeth to do, he does with all his might. The position of Russia in the medical and scientific world is peculiar. Till quite recently the Russians were far behind the nations of Western Europe in every branch of knowledge, and their professors were for the most part Germans, or men educated in Germany. Lately, however, they have shown a remarkable activity and more originality than one would have expected from so imitative a people as the Slavs. In pharmacology and experimental therapeutics especially is this the case, and though those journals published in the German language are naturally the best known here, we hope to glean from the national paper, the *Vratch*, many a fact or hint which may be of service to the practitioner.

**Openings for Young Men.**

"There are no openings for young men" nowadays is the constant and increasing cry from all quarters. Is this cry reasonable or just? We think not, because it arises from the faulty education of those who seek work, and from individual and personal defects of character. The majority of young men who have to earn a living have strange ideas of the meaning of the word "work." Many, like certain Government clerks, usually spend much of their time at the office in watching the clock with a view to rushing away at the earliest possible moment to recreation or amusement of some kind. Nearly every employer has the same tale to tell. Asked if he has any opening for a really good man, he answers, "Certainly, provided he is willing to work, learn, and get results." We fear the "no opening" cry must tend to increase rather than diminish, until the young are made to feel that it is dishonest and dishonourable to accept money in return for services which they never really attempt to render properly at all. Be this as it may, the youths of our day and generation who are really worthy may take courage, because for this class of young people there are, and always will be, openings enough and to spare.

**Londoners and Their Railways.**

Of the many matters which tend to depreciate the health of residents in and around London, railway mismanagement is by no means the least serious. The condition of the carriages on the Metropolitan Railway, and especially of the first-class carriages, is not only frequently disgraceful, but dangerous to health. Dirt of all kinds is allowed to accumulate beneath the seats, whilst the cushions, carpets, windows, and carriages generally are often so filthy as to repel a person entering them from outside. Why is it that the management does not provide for the thorough cleansing of these carriages at least once in the twenty-four hours? It is small wonder when to the impurities of the atmosphere are added the filth dangers referred to, that the travelling public are found in increasing numbers to prefer the omnibus to the railway. Passengers are certainly wise to do so in the interests of their own health. We have often shown the dangers attending catching trains, but another danger of equal extent may arise from the vexation and excitement caused by delays on the railway itself. The unpunctuality of the trains on the South-Eastern Railway at the present time is said to be rapidly depreciating house property throughout South-East London. Yet the unpunctuality complained of could be avoided by a very simple rearrangement of the traffic. If instead of allowing every train to go into Cannon-street all trains were to be stopped at London Bridge, except those going direct to Charing Cross, an effectual remedy would be supplied. Passengers for Cannon-street could then be conveyed to and from London Bridge by a local service of trains in and out of Cannon-street Station, which should, of course, monopolise these lines entirely. It is monstrous to remember that the arrangements for the traffic on this portion of the railway are practically identical with what they were twenty years ago. At any rate, unless or until these railways display a better regard for the health and comfort of Londoners than they do at present, they will be wisest who avoid using this mode of conveyance in and around London as much as possible.

